# The Effect of Developing a Tunnel across a Highway on the Water Quality in an Upstream Reservoir Watershed Area—A Case Study of the Hsuehshan Tunnel in Taiwan

**DOI:** 10.3390/ijerph9093344

**Published:** 2012-09-19

**Authors:** Guey-Shin Shyu, Bai-You Cheng, Wi-Ta Fang

**Affiliations:** 1 Department of Environmental Management, Tungnan University, No. 152, Section 3, Beishen Road, Shenkeng District, New Taipei city 222, Taiwan; 2 Department of Environmental Resources Management, TransWorld University, No. 1221, Zhennan Road, Yunlin County 640, Taiwan; Email: biyocheng@gmail.com; 3 Graduate Institute of Environmental Education, National Taiwan Normal University, No. 88, Section 4, Ting-chou Road, Taipei 116, Taiwan; Email: wawaf@hotmail.com

**Keywords:** reservoir water quality, Hsuehshan Tunnel, phosphorus, non-point sources pollution

## Abstract

Cities in Taiwan are so dependent on reservoir water that preservation of the upstream reservoir watershed has become a significant public concern. However, due to the high-density development of land, resulting in rapid urban expansion, the construction of tunnels and elevated highways across reservoirs to better utilize the surrounding land has become a global trend. Based on data from long-term observation of the reservoir, this study verifies the difference in water quality before and after the highway construction. The results indicate that the total phosphorus (TP) increased on average 14 μg/L to 36.5 μg/L per annum, and the water quality is expected to require 10 years to recover. During the highway development, the average TP was more than twice the normal level. During summer, the TP level increases 3.1-fold due to rainfall. As indicated by the results, the large-scale land development will harm the long-term preservation of the reservoir’s water quality, and therefore should be avoided.

## 1. Introduction

In Taiwan, due to its short rivers, steep slopes, and high flow rates, drinking water availability is primarily contingent on the use of reservoirs. When the project to protect the water quality of the reservoirs was developed, the government recommended higher quality requirements and environmental protection measures. For example, wastewater caused by civil works projects should be intercepted and processed before draining into the reservoir, and the vegetation of destroyed hillsides should be rebuilt. Monitoring of the water quality required enforcement. However, the passiveness of environmental monitoring rendered assessing the actual environmental effects of the projects difficult [1]. Under such circumstances, there was a significant need for long-term water quality monitoring to clearly understand water quality variations [2]. To verify reliability, other reservoirs with similar conditions were utilized as the control group.

Eutrophication can be defined as the presence of excessive nutrient substances in water, which stimulates biological growth of organisms such as algae and hydrophytes, and thus influences the water supply chain. Nutrient substances are typically derived from urban garbage, industrial wastewater and sewage, agricultural runoffs, forest runoffs, urban and marketplace runoffs, and atmospheric fallout. Nitrogen and phosphorus are the important factors that control nonpoint source pollution [3,4,5,6]. The most effective way to control eutrophication is to prevent nutrient substances from flowing into the water. According to preceding research, nonpoint source pollution is closely related to land use [7,8,9,10,11]. Land use of tunnel construction varies significantly regarding both time and space. To date, a relevant standard calculation formula is still unavailable. Groundwater penetration is quite commonplace during tunnel construction. However, not only does the groundwater penetration damage construction equipment and endanger construction workers, it also displaces a significant quantity of sediment and exacerbates soil erosion, presenting a serious hidden risk to the water quality of the reservoir [12]. In this case, during the construction of the Hsuehshan Tunnel, a sudden spewing of groundwater eroded several tea gardens and increased the likelihood that nutritive salt entered the reservoir.

In Taiwan, the strategy for river pollution control is that point source pollution control takes precedence over nonpoint source pollution control. The upstream watershed is primarily surrounded by ancient forests and small villages. However, there are no countermeasures to manage nonpoint source pollution problems arising from the tunnel’s construction. Research by Chou *et al.* [11] indicates that without a 37% decrease of TP the oligotrophic conditions of the Feitsui Reservoir will be impossible to maintain. The upstream project will aggravate the trophic state of water quality in reservoir downstream.

Using data from long-term monitoring, this study evaluates the long-term effects on reservoir water quality of constructing a highway and tunnel in an upstream reservoir watershed. Considering the few external pollutants and abundant vegetation of the watershed, the effects of the large-scale project can be clearly documented by long-term monitoring. This study attempts to confirm the hidden pollution hazards of the project and determine the length of their influence.

## 2. Materials and Methods

This study categorized the water quality monitoring data of the Feitsui Reservoir as the experimental group and that of the Shihmen Reservoir and Tsengwen Reservoir as the control groups to compare the effects of the construction of highways and the Hsuehshan Tunnel on reservoir water quality.

### 2.1. Overview of the Hsuehshan Tunnel

With a total length of 12.942 km, the Hsuehshan Tunnel, located between Pinglin, New Taipei City, and Toucheng, Yilan County, is the longest tunnel in Taiwan and the fifth longest road tunnel in the World. The tunnel connects the city of Taipei to the northeastern Yilan County. The construction of the main tunnel began in July 1993, and the tunnel opened in June 2006. The Hsuehshan Tunnel is comprised of two main tunnels (with a length of 12,942 m and a diameter of 11.8 m) and one pilot tunnel (with a length of 12,942 m and a diameter of 4.8 m). The pilot tunnel, the lining of which is 30 to 60 cm thick, is used for emergency rescues. 28 transverse walking tunnels, eight vehicle tunnels, and shafts and air vents (including three ventilation stations, three ventilation repeaters, 12 transverse ventilation tunnels, and six airshafts) comprise the exhaust system of the two main tunnels. The Hsuehshan Tunnel boasts the largest group of dual-bore highway tunnels in the World, and its construction cost 640 million USD [13].

**Figure 1:**
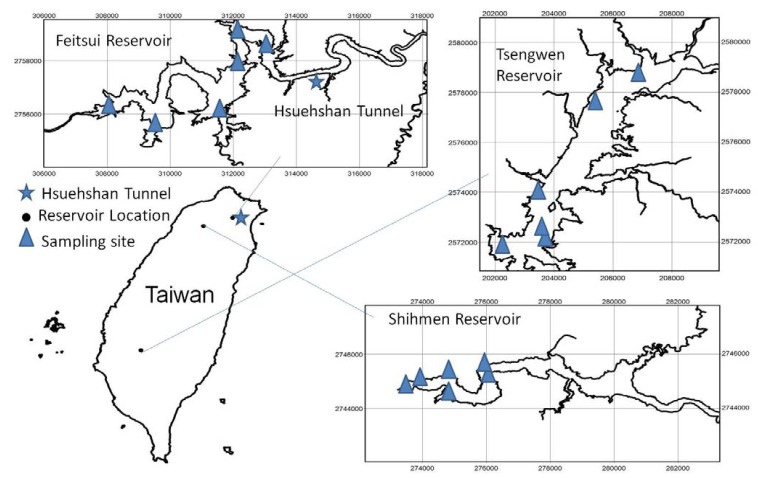
Research area.

### 2.2. Overview of the Feitsui Reservoir, Shihmen Reservoir, and Tsengwen Reservoir

This study primarily discusses the changes in the water quality of the upstream Feitsui Reservoir watershed following the construction of highways and tunnels, while using data on the water quality of Shihmen Reservoir and Tsengwen Reservoir as the control groups. The volume of clean water currently held by the Feitsui Reservoir, which is primarily supplied to people in Taipei and New Taipei City, is 87.1% of effective storage capacity. The upstream watershed of the Feitsui Reservoir has a long history of tea plantations. Due to the favorable soil and water conservation, vegetation is sustained [2,5,6,14]. The Shihmen Reservoir, adjacent to the Feitsui Reservoir, has similar meteorological and hydrological conditions, and the volume of water currently held is 49.8% of the effective storage capacity. Some of the areas in New Taipei City supplied with drinking water by the Shihmen Reservoir overlap with those supplied by the Feitsui Reservoir. The prevailing agricultural practices and sightseeing activities, as well as excessive deforestation of some areas in the upstream watershed of Shihmen Reservoir exacerbate the siltation. Additionally, the increasing water turbidity caused by typhoons have led to a short supply of drinking water. Taiwan’s largest reservoir, the Tsengwen Reservoir, is the major reservoir in the south of Taiwan. The water stored here is primarily used for irrigation, and the current stores are only 13.28 % of its effective storage capacity (shown in Table 1 and Figure 1).

**Table 1:** Reservoir basic information.

**Name**	Feitsui Reservoir	Shihmen Reservoir	Tsengwen Reservoir
**Group**	Experimental Group	Control Group 1	Control Group 2
**Watershed (km^2^)**	303	763.4	481
**Gross Reservoir Capacity (m^3^)**	460 million	390 million	590 million
**Reservoir’s Water Quality**	Mesotrophic	Mesotrophic to Eutrophic	Mesototrophic
**Water Supply Capacity (CMD)**	3.45 million	1.48 million	0.35 million
**Supply Population**	5.5 million	3 million	1.8 million
**Main Function**	Drinking, flood prevention, and electricity generation	Irrigation, drinking, flood prevention, electricity generation, and sightseeing	Irrigation, flood prevention, electricity generation, and sightseeing

### 2.3. Reservoir Water Quality Data

Reservoirs are important source of drinking water in Taiwan, so it is important to build a good reservoir water quality monitoring network, and to analyze water quality changes in different circumstances. The Environmental Water Quality Information Database established by Taiwan’s Environmental Protection Administration (EPA) developed a database of water bodies and water quality monitoring data. This study obtained the reservoir monitoring data from the database, and all data was sampled and tested using the standard methods proposed by the EPA. Chemical analysis of reservoir water quality evaluated the 18 aspects of: temperature, transparency, pH, dissolved oxygen (DO), conductivity, turbidity, suspended solids (SS), hardness, total alkalinity, chemical oxygen demand (COD), ammonia nitrogen, nitrate nitrogen, nitrite nitrogen, organic nitrogen, total phosphorus (TP), phosphates, chlorophyll A, and total organic carbon (TOC). Water sampling is conducted quarterly, and each location is sampled at depths of 0.5 m, 50 m, and 100 m. Sampling has been conducted since 1993 to 2011. There are six water sampling sites in each reservoir with a sampling frequency of four times per year during four seasons. A total of 2,927 sampling and analysis records were obtained for evaluating water quality. In which 1,217 records of Feitsui Reservoir, 767 records of Shihmen Reservoir and 943 records of Tsengwen Reservoir, respectively.

### 2.4. Water Quality Trend Test

The Mann–Kendall test is a non-parametric test which is commonly used to assess the significance of trends in time series such as water quality, stream flow, temperature, and precipitation. We used the Mann–Kendall test to detect whether the trends of the experimental group and control groups occur or not as the variations of water quality in each year turned slightly. This test is can use for making up part of incomplete or missing data. Equations (1) and (2) illustrate the process of calculating S [15,16]:


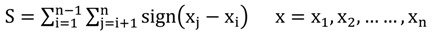
(1)


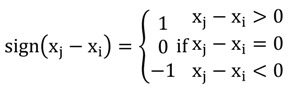
(2)

where S is Kendall score; i is any position less than j, j is position at the jth time, sign is evaluating function. When the value of S is greater or less than ‘0’, and H_0_ is rejected (H_1_: S ≠ 0, at a confidence level of α = 0.05), the time-series trend either rises or decreases. Otherwise, if H_0_ is accepted (H_0_: S = 0, at a confidence level of α = 0.05) there is no correlation between the two variables, with which mean a negligible or nonexistent trend in the time series. This method is proved to be robust to determine trend of time series [17]. The Kendall Package based on R-model (Version 2.6.2) was applied in this study. Missing data were supplemented according to temporal coherence. A positive tou-value is shown as an upward trend, whereas a negative value is shown as a downward trend. The R-model was used to establish matrices from the program for the calculation of tou-values and *p*-values based on various water quality data collected from each year and each site.

## 3. Results and Discussion

### 3.1. Water Quality of Reservoir

The data samples of reservoir water quality between 1993 and 2011 were statistically analyzed, as shown in Table 2. The data are computed as mean value ± standard deviation. 

**Table 2:** Basic statistical analysis of reservoir water quality.

	Temperature	Transparency	pH	DO	Conductivity	Turbidity	SS	Hardness	Total Alkalinity
Experimental Group	21.8 ± 3.8	3.2 ± 1.3	7.25 ± 0.59	7.1 ± 1.7	77.9 ± 12.3	5.85 ± 19.8	4.99 ± 13.9	23.38 ± 8.39	17.85 ± 5.3
Feitsui Reservoir	**COD**	**Ammonia Nitrogen**	**Nitrate Nitrogen**	**Nitrite Nitrogen**	**Organic Nitrogen**	**TP**	**Phosphates**	**Chlorophyll A **	**TOC**
	4.9 ± 4.85	0.10 ± 0.09	0.51 ± 0.19	0.008 ± 0.013	0.34 ± 0.34	26.4 ± 36.0	16.2 ± 36.19	1.7 ± 1.6	1.19 ± 0.64
Control Group 1	20.7 ± 4.4	1.7 ± 0.9	8.05 ± 0.56	7.7 ± 1.9	218 ± 36.0	15.24 ± 64.9	10.78 ± 60.8	94.08 ± 15.74	67.41 ± 15.9
Shihmen Reservoir	**COD**	**Ammonia Nitrogen**	**Nitrate Nitrogen**	**Nitrite Nitrogen**	**Organic Nitrogen**	**TP**	**Phosphates**	**Chlorophyll A **	**TOC**
	5.44 ± 6.29	0.064 ± 0.07	0.36 ± 0.20	0.007 ± 0.019	0.25 ± 0.19	36.8 ± 38.7	24.1 ± 22.20	3.2 ± 3.3	1.2 ± 0.51
Control Group 2	24.8 ± 3.1	1.8 ± 0.8	7.98 ± 0.43	6.3 ± 2.2	279 ± 53.0	8.02 ± 12.0	7.03 ± 13.4	118.45 ± 19.17	105.6 ± 19.8
Tsengweng Reservoir	**COD**	**Ammonia Nitrogen**	**Nitrate Nitrogen**	**Nitrite Nitrogen**	**Organic Nitrogen**	**TP**	**Phosphates**	**Chlorophyll A **	**TOC**
	6.34 ± 5.56	0.069 ± 0.10	0.55 ± 0.29	0.008 ± 0.018	0.28 ± 0.23	30.69 ± 33.9	18.8 ± 23.10	2.6 ± 2.7	1.52 ± 0.57

Units: Temperature (°C), Transparency (m), DO (mg/L), Conductivity (μmho/cm 25 °C), Turbidity (NTU), SS (mg/L), Hardness (mg/L), Total Alkalinity (mg/L), COD (mg/L), Ammonia Nitrogen (mg/L), Nitrate Nitrogen (mg/L), Nitrite Nitrogen (mg/L), Organic Nitrogen (mg/L), TP (μg/L), Phosphates (mg/L), Chlorophyll A (μg/L), TOC (mg/L).

Total phosphorus and phosphate values indicate the level of erosion, and their low values reflect the high water quality of the Feitsui Reservoir. Chlorophyll A indicates a good control of algae, while TOC reveals the water of the Feitsui Reservoir was rarely polluted. The water quality indicators above comprise three assessment perspectives: biological assessment, physical assessment, and chemical assessment. 

### 3.2. Compare the Water Quality of TP

**Figure 2:**
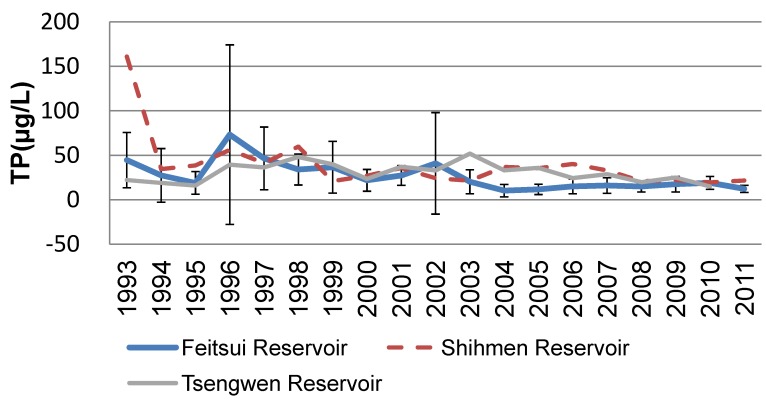
Water Quality Variations of Feitsui Reservoir, Shihmen Reservoir, and Tsengwen Reservoir from 1993 to 2011.

The average TP amounts of both the experimental group and the control group are shown in Figure 2. The highway development began influencing the Feitsui Reservoir in 1993, and the average TP concentration peaked in 1996, exceeding three times the water quality standard of 20 (μg/L) for Taiwan’s reservoirs. The standard deviation of TP reached 101 (μg/L). Such high TP concentrations lead to algae breeding in the reservoir, and thus cause an eutrophication problem. Due to the clean water in the Feitsui Reservoir, the load caused by the highway construction can be alleviated, preventing the downstream area from being influenced. After 2003, the water quality of the Feitsui Reservoir stabilized, with the average TP below 20 (μg/L) and standard deviation of TP below 10 (μg/L). The TP variation tendency indicates that the highway construction has, directly or indirectly, influenced the reservoir’s water quality for nearly 10 years. 

Data from the control group showed a decrease in the TP values of Shihmen Reservoir. According to onsite observations, the prevailing agricultural practices and excessive deforestation in the upstream area have led to serious sand accretion and significant water quality variation. As shown in Figure 2, the water quality variation of the control group differs from that of the experimental group, and therefore, climate can be eliminated as a cause of water quality variation. Table 3 revealed temporal variations in Feitsui Reservoir, Shihmen Reservoir, and Tsengwen Reservoir from six sampling sites each one according to the long-time observation water quality data. All of the monitoring readings showed a positive impact from construction impact. Trend of TP represented a decrease of up to 0.497 in the tou-value (*p* < 0.01) at Feitsui reservoir. And the same situation in Shihmen reservoir, the tou-value is 0.462 (*p* < 0.01). This indicates that main effect was a reduction in TP occurs soon after the highway construction was finished.

**Table 3:** Trend of water quality of TP.

	Score	Var (Score)	Denominator	tau	2-sided *p*value
Feitsui Reservoir	–85	817	171	–0.497	0.003
Shihmen Reservoir	–79	817	171	–0.462	0.006
Tsengwen Reservoir	–21	697	153	–0.137	0.449

**Figure 3:**
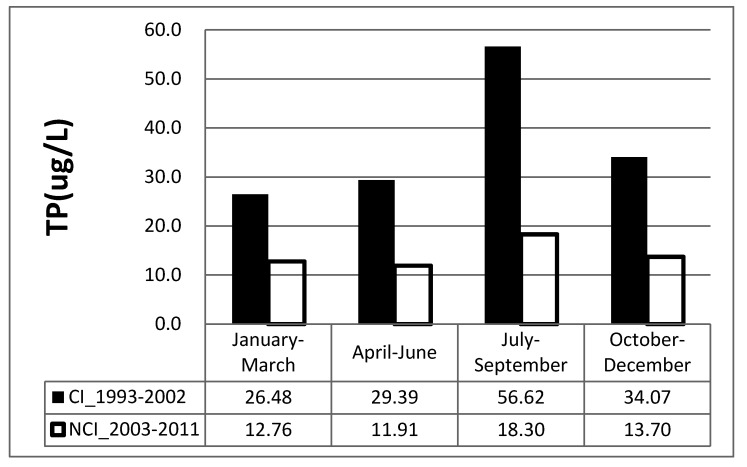
The average TP amount before and after seasonal influence analysis.

According to water quality variation tendencies, 2003, the year the tunnels and highways were constructed, is the cut-off point for the original environment of reservoir water quality. Therefore, a comparison between the TP amount from 1993 to 2002 and that from 2003 to 2011 was made, as indicated in Figure 3. The periods from 1993 to 2002 are construction impact periods and the periods from 2003 to 2011 are non-construction impact periods. Additionally, the TP amount was judged seasonally [18]. During the dry season (January to June), the TP concentration after the construction impact period was low, approximately 2 to 2.5 times the level prior to construction, while during the wet season it was approximately 2.5 to 3 times. That’s indicate that the construction impact on water quality.

### 3.3. Total Phosphorus Mass Balance in the Reservoir

The main sources of nutrients in the reservoir catchment area are rainfall runoff entrained sediment of suspended particles and dissolved substances. When water flows into the river and reservoir (in Feitsui Reservoir), hydraulic water residence time is about 125 days, during this period the nutrient substances will be affected by various biogeochemical reactions, of which adsorption and precipitation are the most significant influences. During the operation of the reservoir, the discharge of water stored will remove some of the suspended particles or dissolved nutrient substances. This can be written as a simple description of the mode, the output + deposition = input. Sedimentation volume in the reservoir can be calculated. For example, about 200,000 kg of phosphorus was washed out into Feitsui Reservoir and the large part accumulated in the sediments. However, water quality models estimate that only 10% of phosphorus content was significantly underestimated in the past. Of phosphorus supply volume cannot reach a stable balance makes the whole environment, therefore, non-point source pollution is undervalued long-term. To explain this status, although phosphorus in sediments has a trend to release the pollutant into water bodies, this driving force is limited by a variety of other hydro activities, such as chemical, biological, and so many factors impact the water quality. Cause by observation catchment water quality not in rainy day. This leads to a large estimation error by commonly used estimation models. Therefore, this study suggests that developments upstream of the reservoir will make for a high potential for pollution of the water of the reservoir.

## 4. Conclusions

In the field of water science, numerous variable environment factors make it enormously difficult to select the experimental group and the control group. A complete understanding of the onsite environment and a significant volume of monitoring data are required to interpret the phenomena. The foundation of this study was the planned monitoring of water quality to establish the variation tendency of water quality, and thereby, serve as a reference for formulating a water pollution prevention strategy. In this study, due to the construction of highways in the upstream watershed, as well as the Hsuehshan Tunnel, the output of nonpoint source pollution rose twice the normal level, and the TP concentrations have also increased over the past ten years. Therefore, this study recommends that the project in the reservoir’s water resources protection area be developed cautiously as it could cause long-term water quality problems.
